# The Consumption of Raw Goat Milk Resulted in TBE in Patients in Poland, 2022 “Case Report”

**DOI:** 10.3390/pathogens12050653

**Published:** 2023-04-27

**Authors:** Angelina Wójcik-Fatla, Joanna Krzowska-Firych, Krzysztof Czajka, Joanna Nozdryn-Płotnicka, Jacek Sroka

**Affiliations:** 1 Department of Health Biohazards and Parasitology, Institute of Rural Health, Jaczewskiego 2, 20-090 Lublin, Poland; 2 Infectious Diseases Clinic, Institute of Rural Health, Jaczewskiego 2, 20-090 Lublin, Poland; firychdr@poczta.onet.pl (J.K.-F.); krzysz.czajka@gmail.com (K.C.); j.m.nozdryn@gmail.com (J.N.-P.); 3 Department of Parasitology and Invasive Diseases, National Veterinary Research Institute, Aleja Partyzantów 57, 24-100 Puławy, Poland; jacek.sroka@piwet.pulawy.pl

**Keywords:** tick-borne encephalitis virus, goat milk, diphase milk fever, TBEV, alimentary route, unpasteurized milk, non-vectorial transmission

## Abstract

The alimentary route is the second most important route of tick-borne encephalitis infection. In Poland, the last TBE case due to the consumption of unpasteurized milk or dairy products of infected animals was recorded in 2017 as the fourth documented outbreak of TBEV infection in the country. In this study, two patients infected with TBEV through consumption of unpasteurized goat’s milk from one source are described from a cluster of eight cases. In August and September 2022, a 63- and 67-year-old woman were hospitalized at the Infectious Diseases Clinic of the Institute of Rural Health (Lublin, Poland). The patients denied been recently bitten by a tick, and neither had been vaccinated against TBEV. The disease had a biphasic course. In the first case, the patient suffered from a fever, spine pain, and muscle weakness and paresis of the lower left limb. The second patient suffered from fever, vertigo, headaches, abdominal pain, and diarrhoea. The results of IgM and IgG antibodies were positive in both cases. After three weeks hospitalization, the patients were discharged in good condition. In one case, slight hearing impairment was observed. Vaccination and avoiding the consumption of unpasteurized milk remain the most effective ways to prevent tick-borne encephalitis.

## 1. Introduction

The etiological factor of Tick-borne encephalitis (TBE), also known as early summer or spring–summer encephalitis, is a virus belonging to the *Flaviviridae* family and the Flavivirus genus [1]. Endemic areas of TBE include areas of Central and Eastern Europe, reaching the eastern regions of Asia and Japan [2]. The first clinical reports of the disease, corresponding to the description of tick-borne encephalitis, originated from Austria in the early 1930s. Isolation of the virus responsible for the disease, then known as Central European Encephalitis (CEE), occurred after 1948. In the meantime, similar cases were reported in eastern Russia, where the disease was referred to as Russian Spring-Summer Encephalitis. In 1937, Russian scientists isolated the virus both from the blood of an infected patient and from a tick belonging to the genus *Ixodes*, the bite of which was the direct cause of infection [1,3].

In European countries, tick-borne encephalomyelitis virus infection is one of the most dangerous and potentially fatal infections of the central nervous system [4]. The course of infection, complications after the disease, and mortality depend on the type of virus, of which three closely related subtypes have been distinguished: European, Siberian, and Far Eastern [5]. In Eurasia, the number of registered cases varies from 5000 to 12,000 per year, depending on the country [6]. The highest percentage of cases per year, reaching several thousand, is recorded mainly in Russia, while in European countries, these values usually do not exceed several hundred cases per year [7]. According to the annual reports “Infectious diseases and poisonings in Poland” conducted by the National Institute of Public Health—National Institute of Hygiene (Warsaw, Poland), the number of all cases of tick-borne encephalitis in Poland usually does not exceed 300 per year, with an average of 0.7–0.9 per 100,000 inhabitants [8].

The occurrence of natural endemic foci of the TBE virus is possible due to the circulation of the pathogen between its vector (ticks) and the reservoir, which are vertebrates (mainly rodents) [9]. In Europe, the main role as a competent vector of many dangerous pathogens (including tick-borne encephalitis virus) is played by the common tick species *Ixodes ricinus*. The average percentage of *Ixodes ricinus* tick infections with TBEV ranges from 0.1 to 2.5%, depending on the gender and developmental stage of the tick [10]. Two other flaviviruses transmitted by ticks from the *Ixodes* genus (*I. scapularis* and *I. cookie*) belong the Powassan virus (POWV), which is responsible for infections in North America [11]. 

Apart from virus transmission by tick bite, the second route of infection is the alimentary route, which is responsible for about 1% of all TBEV cases; however, in different countries, infections due to the consumption of unpasteurized milk or dairy products of infected sheep, goats or cows can exceed 20% [12]. Following one of the most recent systematic reviews written by Martello et al., other non-vector transmission of tick-borne encephalitis virus is possible, including infection through handling contaminated materials, blood transfusion, and solid organ transplants [13]. These routes of transmission are extremely rare, similarly to cases of aerosol infections which have been reported sporadically in the literature [14].

In Poland, the last cases of milk fever were recorded in 2017 as the fourth documented outbreak of TBE virus infection, almost 27 years after the small epidemics caused by the consumption of goat milk in 1995 and 1996 [15].

In this publication, two cases of patients are described as infected with tick-borne encephalitis virus by consumption of unpasteurized goat’s milk, diagnosed, and treated in the Infectious Diseases Clinic of the Institute of Rural Health (Lublin, Poland) in 2022.

## 2. Case Presentation

In August and September 2022, two patients from a cluster of eight cases with a diagnosis of tick-borne encephalitis from the gastrointestinal tract were hospitalized at the Infectious Diseases Clinic of the Institute of Rural Health (Lublin, Poland). Both patients were not vaccinated against TBEV.

### 2.1. Case 1

The patient was a 63-year-old woman with no medical history. The patient denied having been recently bitten by a tick. It was established that she drank unpasteurized goat’s milk a few days before the onset of symptoms. According to the patient, all eight people who drank the same milk developed symptoms. On admission, she reported a fever of 38 °C, pain in the spine and lower left limb, and muscle weakness and paresis of this limb. The history revealed that the disease had a biphasic course. The first symptoms occurred a few days after the consumption of unpasteurized goat milk. The patient reported fever, lumbosacral pain, and sweating. The symptoms occurred 16 days before hospitalization, lasted around five days, and then regressed. The second phase occurred after seven days. 

In the blood test results performed during hospitalization, we observed anaemia, elevated D-dimers (1006 ng/mL) and hyponatraemia. Neurological examination revealed pain and limitation of neck movement with the absence of meningeal symptoms. The patient also presented weakness of the proximal muscles of the left lower limb without symptoms of pyramidal tract damage and without superficial sensation disorders.

On admission, a lumbar puncture was performed. The results of cerebrospinal fluid examination during hospitalization are presented in Table 1. Plain computer tomography brain imaging was normal. The results of cultures of blood and cerebrospinal fluid were negative.

The diagnosis was based by the detection of specific IgM and IgG serum antibodies against tick-borne encephalitis (TBE) and performed by ELISA test using EuroImmun Medizinische Labordiagnostika AG (Lübeck, Germany) kits: Anti-TBE Virus ELISA (IgG) and Anti-TBE Virus ELISA (IgM), both with IVD (approved for in vitro diagnostics). Whole blood was collected from patients at the Infectious Diseases Clinic and immediately transported to the Medical Diagnostic Laboratory of the Department of Health Biohazards and Parasitology (both in the Institute of Rural Health, Lublin, Poland). The tests were performed according to the manufacturer’s recommendations for semiquantitative assessment with serum on the day of blood sampling. Optical density was measured at a wavelength of 450 nm and a reference wavelength of 620–650 nm with the use of MultiskanTM FC Microplate Photometer (ThermoFisher Scientific, Waltham, MA, USA). Results were evaluated by calculating the ratio factor: control or patient sample extinction to calibrator extinction. The results of both IgM and IgG antibodies of the patient were positive (ratio: 3.49 and 2.05, respectively; at the cut-off range ratio: 0.8–1.09). 

During hospitalization, we observed fever above 38 °C with chills, muscles weakness, and pain, followed by sleepiness, headaches, nausea, and vomiting. Symptomatic treatment was carried out. After seven days of hospitalization, the symptoms gradually subsided and the general condition of the patient improved. The patient was discharged in good condition after 18 days of hospitalization.

### 2.2. Case 2

The patient was a 67-year-old female treated for hypothyroidism. The first symptoms occurred seven days after the consumption of unpasteurised goat milk. A tick bite was not confirmed in the medical interview.

The clinical course was biphasic. The patient suffered from fever, vertigo, headaches, abdominal pain, and diarrhoea, and the symptoms resolved within a few days without therapy. The next phase occurred after two weeks, with fever, headaches, vertigo, shaking hands, periodically pain in the calf muscles, and excessive sleepiness. On admission, the patient complained of headaches, vertigo, fatigue, and shaking hands. She did not have a fever. On physical examination, the Neri’s test was performed, which is used for the detection of nerve root irritation in the lumbosacral area and, in particular, for the diagnosis of a lumbosacral radicular syndrome. Physical examination revealed photophobia, ataxia, hearing impairment, and muscle wasting. 

The blood tests performed on admission showed elevated D-dimers and lower TSH concentration. The results of cerebrospinal fluid examination during hospitalization are presented in Table 1. The culture of CSF was negative. The results of both IgM and IgG antibodies were positive (ratio: 5.58 and 3.35, respectively; at the cut-off range ratio: 0.8–1.09). 

Plain computer tomography brain imaging was normal. The patient was hospitalized for 21 days, and on discharge slight hearing impairment was observed. She was referred for an audiological examination.

## 3. Discussion

The patients described in this study were infected by the consumption of milk from an infected goat owned by a private person who did not run a business in the field of food production of animal origin. Nevertheless, the Regulation of the Minister of Agriculture and Rural Development on veterinary requirements for the production and products of animal origin intended for direct sale, in the case of raw milk, does not indicate the need to perform tests for the presence of tick-borne encephalitis virus. Raw milk intended for direct sale is obligatory tested at least once a month but only for the presence of bacteria and somatic cells [16]. The regulations also state that raw milk should come from healthy animals, without visible signs of disease and that do not show signs of infectious diseases transmitted to humans by food; however, studies on livestock, such as sheep, goats, and cows, show that infection with Tick-borne encephalitis virus is usually asymptomatic [17,18], and cases of symptomatic infection are very rare [19]. In addition, the presence of TBE virus in sheep, goat, and cow populations tends to be focal; therefore, seroprevalence can vary widely from 0% to 40%, depending on the country or region. Conducting tests within individual herds could increase the probability of detecting smaller outbreaks of TBEV infection [20].

Scientific studies have shown that infected goats can shed the virus in their milk for up to 25 days [21]. Research conducted at the Institute of Rural Health in Lublin confirmed the presence of TBE virus in sheep’s milk at 22%, in goat’s milk at 20%, and in cow’s milk at 11%; however, it should be noted that the milk samples came mainly from animals grazing on pastures, where the risk of tick bites is higher than in farm breeding [22]. Despite this, most milk sample testing focuses on specific food outbreaks rather than epidemiological screening; therefore, the percentage of positive results will vary in different countries [23]. In Lithuania, the percentage of positive results of bulk-tank milk from sheep and goats has been confirmed at the level of 4.3% and 4.5%, respectively [23]. 

The virus is sensitive to temperature changes, hence, after drying or pasteurization, it loses its infectious properties, completely preventing infection with TBE virus via the alimentary route. The decrease in virus infectivity in milk is observed after 30 min at 60 °C, while pasteurization at 72 °C for 10 s causes complete inactivation of the virus [24]. Storing unpasteurized milk at 4 °C also does not protect against infection, as the virus retains its infectious properties for at least three days at this temperature [25]. The TBE virus remains active in environments with a low pH [26], is not instantly inactivated in the stomach, and can retain its infectious properties for up to two hours in dairy products from the moment of their consumption [27]; therefore, the virus can be transmitted through the gastrointestinal tract and, additionally, can multiply in the cells of the intestinal epithelium [28]. Infection through the consumption of unpasteurized milk can also lead to a shortened incubation period of two or three days [29] compared to infection caused by a tick bite, where the incubation period varies from seven to 14 days [30].

In our study, the first symptoms appeared up to seven days after drinking raw goat’s milk. In the case of alimentary infection, clinical courses could be monophasic or biphasic, with flu-like symptoms and high temperature in the first and second phases, respectively [31]. The history revealed that both described cases had a biphasic course similar to the case report of family outbreak in Austria [32]. In 2019, a cluster of five patients hospitalized due to tick-borne encephalitis after consuming unpasteurized goat milk was reported in Croatia [33]. In all the cases described above, the patients were not vaccinated against TBEV. In our study, PCR tests for the presence of the virus in blood and serum samples were performed, but negative results were received because both patients were hospitalized after the first symptoms occurred [34]. According to a Polish team of experts (infectious disease doctors and epidemiologists), the detection of IgM and IgG antibodies in serum or cerebrospinal fluid using an enzyme-linked immunoassay (ELISA) enables the diagnosis of TBE in the neurological phase and is the diagnostic method of choice. On the other hand, the detection of viral nucleic acid is not applicable in the late neurological phase of the disease due to too low sensitivity and specificity [35]. In both patients described in our study, IgM and IgG antibodies were present in the blood serum.

Natural infection with TBEV confers immune protection against reinfection due to long-term or, in some cases, life-long persistence of serum IgG antibodies [36]; however, the mortality rate due to TBEV infection in Europe and all Eurasia rises by 5% and 15–20%, respectively [37]. In addition, the rate of complications after TBEV infection is estimated at about 20–50%. The most common complications include paralysis and paresis of cranial nerves, multi-neural damage with paralysis of various muscle groups, flaccid paralysis of limbs, as well as speech and gait disorders and nystagmus resulting from damage to the cerebellum. In some cases, concentration, attention, and memory disorders may occur [38]. One of the described patients (Case 2) developed gait disturbance due to cerebellar dysfunction.

The clinical course in our patients was stable with no symptoms of central nervous system involvement. In both cases, CSF examination was performed with CT examination which excluded meningoencephalitis with no brain involvement. Both patients were consulted by a neurologist. There is no specific treatment for TBE, and symptomatic therapy is recommended. Only patients with severe encephalitis should be empirically treatment with intravenous acyclovir and antibiotics until herpes and bacterial infections are ruled out, but our patients did not meet these criteria [39]. Similarly, for the previously mentioned Powassan virus infection, treatment is usually supportive, but in the one described case of *Borrelia burgdorferi* and POWV co-infection, improvement of the patient’s condition was achieved by empiric intravenous immunoglobulin (IVIG) inclusion in the treatment [11]. In our study, both patients denied being bitten by ticks, and the symptoms were strictly related to the consumption of raw goat milk. Based on the history and the fact that Lyme disease, anaplasmosis, and other tick-borne pathogens are not transmitted by foodborne route, these diseases were not included in the differential diagnosis.

The clinical course in patients with food-borne TBE differs from that in tick-bitten patients. The incubation period is shorter in patients infected by the alimentary route. Data from the literature indicate that the biphasic form of TBE is more common in milk-borne cases and after tick transmission, occurring only in 20–30% of infected patients, and in the group of tick-associated TBE, the neurological manifestation observed in the second phase is more severe and includes meningitis, meningoencephalitis, and meningoencephaloradiculitis. The majority of patients with milk-borne infection recover with no neurological sequelae [12].

The most effective way to prevent tick-borne encephalitis is to use vaccines that provide full protection against this virus. In Poland, according to the Annual Reports “Vaccinations in Poland”, the number of people vaccinated since 2015 (27,849) had more than doubled by 2021 (67,527) [40]. Obligatory vaccinations apply only to selected occupational groups, mainly foresters exposed to frequent tick bites. In Poland, as is in most countries in Europe, there are no legal regulations regarding vaccinations against TBEV, but as of 2021, recommendations prepared by a team of experts could help in controlling infections [41]. In the case of foodborne infections, the only way is to avoid eating unpasteurized milk and its products. It would also be helpful to document cases of non-vectorial transmission of TBE other than by the alimentary route [13].

According to scientific reports, since 2015 there has been an increasing number of cases of TBE infections. The factors contributing to the increase in the incidence of TBE include climate warming, the dynamics of socio-economic changes, and the increase in the number of people traveling to endemic areas of tick-borne encephalitis [42].

Tick-borne encephalitis remains the only tick-borne disease for which an effective vaccine is available. Vaccination and educational activities offer a real chance to reduce the number of cases of TBE virus infection.

## Figures and Tables

**Table 1:** Cerebrospinal fluid parameters of hospitalized patients.

Patient	Parameters					
	Colour and Transparency	Cytosis (0–1 Cells/µL)	Glucose (50–80 mg/dL)	Protein (15–60 mg/dL)	Lymphocytes (%) (40–80)	Non-Apelt Test
Patient No. 1	aqueous, clear	39	54	87	78	negative
Patient No. 2	aqueous, clear	62	42	50	93	negative

## Data Availability

All data generated or analysed during this study are included in this published article and are available from the Clinic of Infectious Diseases (Institute of Rural Health, Lublin, Poland).
